# Gain-of-function p53 mutants have widespread genomic locations partially overlapping with p63

**DOI:** 10.18632/oncotarget.447

**Published:** 2012-02-22

**Authors:** Elena Martynova, Silvia Pozzi, Valentina Basile, Diletta Dolfini, Federico Zambelli, Carol Imbriano, Giulio Pavesi, Roberto Mantovani

**Affiliations:** ^1^ Dipartimento di Scienze Biomolecolari e Biotecnologie. Università degli Studi di Milano, Milano, Italy; ^2^ Dipartimento di Biologia, Università degli Studi di Modena e Reggio Emilia, Modena, Italy

**Keywords:** mutant p53, p63, keratinocytes

## Abstract

p53 and p63 are transcription factors -TFs- playing master roles in the DNA-damage response and in the development and maintenance of pluristratified epithelia, respectively. p53 mutations are common in epithelial tumors and HaCaT keratinocytes harbor two p53 alleles -H179Y and R282Q- with gain-of-function (GOF) activity. Indeed, functional inactivation of mutp53 affects the growth rate of HaCaT. We investigated the strategy of mutp53, by performing ChIP-Seq experiments of mutp53 and p63 and analyzed the transcriptome after mutp53 inactivation. Mutp53 bind to 7135 locations *in vivo*, with a robust overlap with p63. *De novo* motifs discovery recovered a p53/p63RE with high information content in sites bound by p63 and mutp53/p63, but not by mutp53 alone: these sites are rather enriched in elements of other TFs. The HaCaT p63 locations are only partially overlapping with those of normal keratinocytes; importantly, and enriched in mutp53 sites which delineate a functionally different group of target genes. Our data favour a model whereby mutp53 GOF mutants act both by tethering growth-controlling TFs and highjacking p63 to new locations.

## INTRODUCTION

p53/p63/p73 are a family of transcription factors -TFs- that share a conserved DNA-binding domain and a similar DNA target sequence in promoters and enhancers [1-4]. p63 is involved in the development and maintenance of the skin and of pluristratified epithelia. The major isoform present in the skin -DeltaNp63alpha- is essential for development of ectoderm and stratification, through activation of the epithelial cell adhesion program, and it plays a major role in maintaining the proliferative potential of stem cells [3]. p63 is overexpressed in many epithelial tumors, notably Squamous Cell Carcinomas, SCC [4]. The risk of developing SCC is directly related to UV exposure and mutations in p53 are clearly a predisposing factor: indeed UVB “signature” mutations were reported [5]. HaCaT cells are immortalized, non tumorigenic keratinocytes with mutant p53 alleles, R282Q and H179Y [6], which are typical UVB signatures [7]. In this system, the p53 pathway is functional in response to UVB irradiation, in terms of cell cycle block and induction of apoptosis [8, 9]. These cells also express large amounts of DeltaNp63alpha.

Abolition of the p53 powerful tumor suppression functions is an important step in cancer progression, and the location of hotspot mutations in residues important for DNA-binding provided a conceptual framework pointing at a loss-of-function mechanism. However, there is now strong genetic evidence that some p53 missense mutants are pro-active in tumor progression and metastasis formation [Reviewed in 10, 11]. The issue of the interplay between mutp53 and p63/p73 is quite relevant, because genetic experiments suggest a complex role of p63 isoforms in transformation [4], and p53 missense mutants, including those produced by HaCaT alleles, have an increased affinity for p63/p73 [12-14, reviewed in 15]. In addition, the three family members are linked through a microRNAs-based circuit [16]. Two gain-of-function -GOF- mechanisms have been suggested: the first posits that an excess of mutp53 interferes with p63/p73 function, by inhibiting DNA-binding following a stimulus, or forming inactive aggregates [17]. In the second, mutp53 do reach specific DNA targets, either through protein-protein interactions with other TFs, such as NF-Y, E2F1, NF-KB and VDR [18-21], or *via* p63-guided interactions [22, 23]. Note that “indirect” promoter recruitment of a TF by interactions with another TF bound to its element was first described for the Estrogen Receptor on Fos/Jun sites two decades ago [24], and further detailed for many other TFs ever since. The relative “weight” of the two scenarios is unclear. To examine this, we decided to explore mutp53 *in vivo* DNA-binding and functional activities, and relate it to p63 locations in HaCaT cells.

## RESULTS

**Figure 1:**
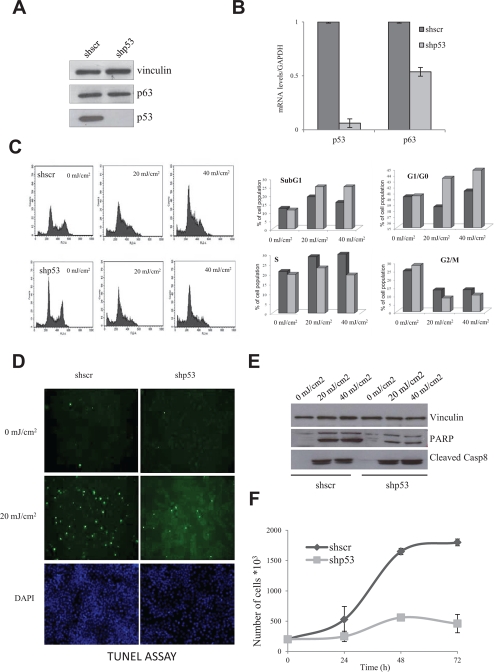
Effect of functional inactivation of mutp53 in HaCaT cells (A) mutp53 was inactivated by transduction of the HaCaT cells by lentiviral vector expressing shp53 and vector expressing scramble shRNA served as a negative control. After puromycine selection clones were selected, pooled and protein levels of mutp53 and p63 were analyzed by Western blot. Vinculin was used as a loading control. (B) mRNA levels of mutp53 and p63 were subsequently controlled by qPCR. Normalization of cDNA templates was achieved by GAPDH quantification. (C) Inactivation of mutp53 in HaCaT cells leads to the modest variation of cell cycle progression. The medium of HaCaT cells was replaced by PBS and the cells were exposed to 20 mJ/cm^2^ and 40 mJ/cm^2^ concentration of UVB light. After UVB treatment PBS was replaced to growth medium and after 12h of incubation at the standard conditions cells were harvested and cell cycle progression was analyzed by FACS. PBS-treated cells without UVB treatment served as negative control. (D) mutp53 deprived HaCaT cells are less sensitive to the apoptosis. shp53 and shscramble HaCaT cells were treated with UVB irradiation and degree of apoptosis was measured by TUNEL assay (E) as well as PARP activation and Caspase 8 cleavage were controlled by Western blot. (F) mutp53 affects significantly the growth properties of the cells. Cell growth rates of shp53 and shscramble HaCaT were compared by direct counting of viable cells.

Mutp53 proteins are highly expressed in HaCaT cells [15], as it often the case with other missense mutp53 alleles: we inactivated the two alleles by stably expressing an shRNA under puromycin selection, in parallel with a control scramble shRNA. The p53 shRNA targets the DNA-binding domain, hence it is aimed at all possible isoforms of p53 [25]. Clones were selected, pooled and mRNA and protein levels of mutp53 and p63 controlled by qRT-PCR and Western blot: Fig. 1A shows that mutp53 mRNA levels are substantially decreased, the protein levels essentially abolished (Fig. 1B). p63 mRNA was modestly decreased. Note that a similar inactivation experiment attempted with p63 shRNAs repeatedly failed to yield colonies, likely because of the key role of p63 in HaCaT survival and growth (EM, RM, unpublished). We studied a few parameters of mutp53-expressing and non expressing HaCaT, before and after UVB irradiation. As shown in Fig. 1C, we noticed a modest variation in cell cycle progression: increase in subG1 and G1 cells, and a decrease in G2/M in mutp53-depleted cells. The degree of apoptosis, as measured by TUNEL assays (Fig. 1D), PARP activation and Caspase 8 cleavage (Fig. 1E) was lower in the absence of mutp53, but still present. Thus other mechanisms compensate for the lack of mutp53 to drive a DNA-damage response. The most notable difference, however, was the growth rate, as curves were flatter in HaCaT deprived of mutp53 (Fig. 1F). These data confirm that mutp53 are indeed GOF alleles contributing to cellular growth.

To investigate the GOF mechanism, we performed analysis of p63 and mutp53 binding to genomic locations by ChIP-Seq experiments, using the DO1 monoclonal, recognizing the N-terminal domain of p53, and a polyclonal against p63. We identified 7135 peaks of mutp53 and 3421 of p63 in HaCaT cells, defined as areas with a significant enrichment in the IP with respect to the corresponding genomic region of Input DNA controls run in parallel (see Methods). The lists of locations and transcription Units are in Supplementary Tables 1 and 2, respectively. Notorious p63 targets such as the p21 and MDM2 promoters were among the positives (See below). We validated the data by using different antibodies, the Ab7 p53 polyclonal, which recognizes all p53 isoforms, and anti-63 4A4 monoclonal (Supplementary Fig. 1). Some of the positive regions in ChIP-Seq were monitored by qPCR: with the exception of FANCi, the other targets were enriched. We noticed that some targets scoring positive only for p63 in ChIP-Seq were also somewhat enriched with Ab7, suggesting that we might be underscoring the overlap of the two TFs, possibly because DO1 is unable to pick up the shorter p53 isoforms present in HaCaT cells [25]. Finally, p63 targets previously identified as functionally important, such as KLF4, Notch1, TP63, DLX3/4 and JAG2, among others, scored positive for p63 and mutp53 (Supplementary Fig. 2).

**Figure 2:**
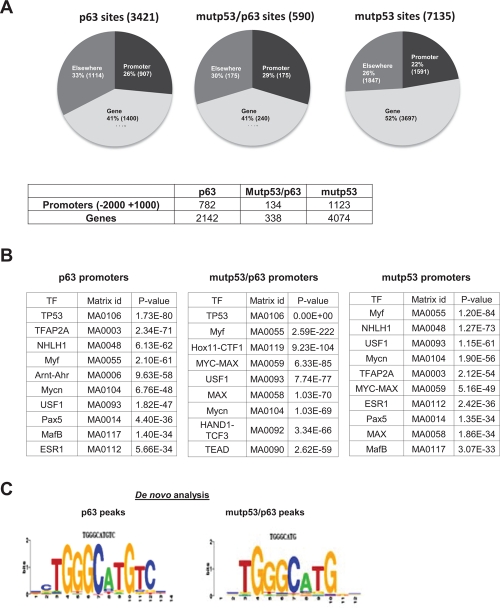
ChIP-Seq analysis of p63 and mutant p53 binding in HaCaT keratinocytes (A) Distribution of the site position for mutp53 and p63 as well as the overlap between p63 and mutp53 positive peaks are present (UCSC genes http://genome.ucsc.edu/). Also the number of promoters or genes, positive for at least one peak of mutp53 or p63 or both is indicated. (B) Evaluation of TFBS enrichment in the of p63^+^, mutp53^+^ and p63/mutp53^+^ promoters using Pscan software. (C) Analysis of p63^+^ and p63/mutp53^+^ binding site sequences by *de novo* motif discovery performed using the Weeder tool (26).

We therefore felt confident to analyze the ChIP-Seq locations and found the expected overrepresentation in promoters, from −5000 to +1000 of the Transcriptional Start Sites (TSS): Fig. 2A shows that 1591 mutp53 and 907 p63 sites reside in promoters, and 3697 and 1400, respectively, in the body of RefSeq genes. We characterized the overlap between positive peaks, and found that 19% of p63 peaks overlap with mutp53 in the promoter (175 out of 907) and 17% in the body of genes (240 out of 1400).

Next, we evaluated the enrichment of TFBS in the p63^+^, mutp53^+^ and p63/mutp53^+^ peaks with a width of 150 bp from the center with the Pscan software [26] (Fig. 2B), using as background a set of 10.0000 sequences of the same size, chosen at random from genomic regions annotated either as “promoter” or “enhancer”. It is apparent that a consensus p53/p63RE is at the top of the list in p63^+^ and p63/mutp53^+^ peaks, but not in mutp53^+^, where sites of other TFs predominate: E boxes -variously termed NHLH1, Myf, Mycn, USF1, MYC-MAX, MAX- ESR (nuclear receptors), AP2. This is an indication that p63, either alone or with mutp53, recognizes its own site, whereas mutp53, in the absence of p63, binds DNA through sequences recognized by other TFs. We then used the Weeder software [27] to perform *de novo* motif discovery in the peaks of the three cohorts, in promoters, genes or elsewhere: Fig. 2C shows that a TGGGCATGTC sequence clearly emerged in p63^+^, containing a perfect p53/p63 consensus (underlined), with additional information on the flankings; a similar sequence, lacking the CC at the 3' end, is recovered in p63/mutp53^+^ locations; in mutp53^+^ peaks, instead, the variety of underlying sequences prevented the emergence of a clear consensus by *de novo* analysis, confirming the underlying presence of several unrelated TFBS.

**Figure 3:**
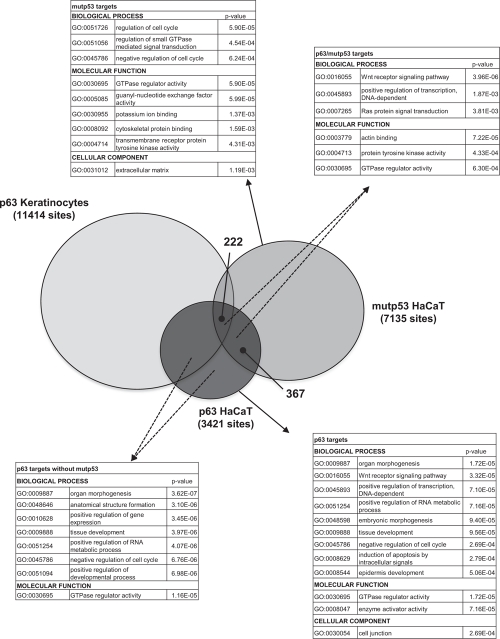
p63 and mutant p53 binding in normal and HaCaT keratinocytes The comparison of p63 and mutp53 DNA binding sites in HaCaT cells and p63 DNA binding sites in PHK. 222 sites were common for p63, mutp53 in HaCaT and p63 in PHK, but the comparison also revealed other 367 sites that were shared by p63 and mutp53 just in the HaCaT cells. Gene Ontology analysis for p63^+^, mutp53^+^, p63^+^/mutp53^+^ p63^+^/mutp53^−^ in HaCaT keratinocytes is present.

A large number of genomic locations of p63 were recently reported in primary keratinocytes -PHK- using the same antibody employed here [28]. We analyzed the data of PHK and HaCaT and found that a substantial number -50%- of HaCaT locations are missing in primary keratinocytes (Fig. 3). We assessed the number of mutp53 peaks in the two populations and found some skewing: 222 were in the PHK common sites, and 367 in the HaCaT-only cohort, suggesting that the presence of mutp53 alters p63 binding to a subset of sites bound in normal keratinocytes. Gene Ontology analysis retrieved terms such as *organ morphogenesis*, *tissue and epidermic development* and *positive regulation of transcription* in p63^+^ devoid of mutp53 (Fig. 3); the same terms were present in the larger p63^+^ cohort, with the addition of *Wnt signaling* and *induction of apoptosis*. Terms related to *signal transduction* and *cell cycle* were prevalent in mutp53^+^ genes (Fig. 3). Specifically, the mutp53/p53+ sites were enriched in terms of *Wnt signalling* and other metabolic terms in the molecular function analysis, such as *actin binding, Tyrosine Kinase* and *GTPase activity*. In addition to previously characterized targets (Supplementary Figure 3a, b), families of targets worth mentioning are Wnt genes -Wnt4, Wnt7a, Wnt9a, Wnt10a- and Retinoic Acids Receptors, RARα, RARγ and RXRα (Supplementary Fig. 3c, d). Both p63 and mutp53 are present at multiple locations of the large cluster of keratin genes on chromosome 17, particularly in a conserved region at 3', overlapping with positive epigenetic marks (Supplementary Fig. 3e). In general, the p63 and mutp53 locations overlap with those of histone post-translational modifications (Supplementary Fig. 3): although firm conclusions are difficult to make because of the difference in the cellular contexts of the ChIP-seq profiles, this is a further indication that the sites identified here are functionally relevant. From this set of data, we conclude that p63 binding in HaCaT is different from PHK, in part due to the coresidency of mutp53, and that the latter recognizes a large set of functionally distinct groups of genes independently from p63.

**Figure 4:**
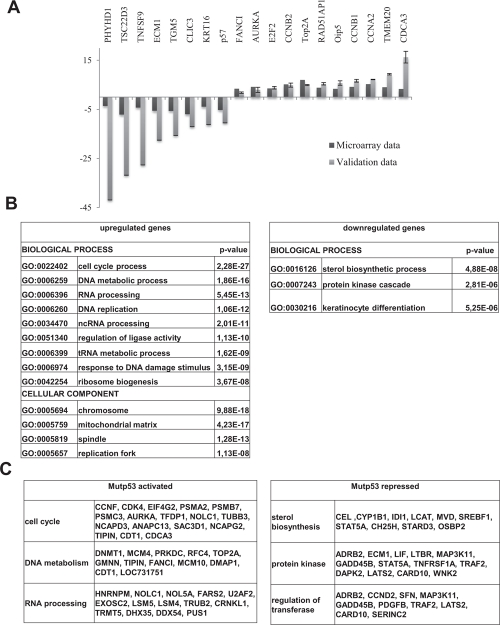
Profiling of mutp53 inactivated HaCaT cells (A) Validation of microarray data by qPCR. (B) GO analysis: for the up-regulated genes cell cycle, as well as DNA and RNA metabolisms and response to DNA-damage categories were retrieved with significant p-value, while for down-regulated genes sterol biosynthesis and keratinocyte differentiation categories were obtained even if with the less significant p-value. (C) Examples of the genes that are up-regulated and down-regulated by mutp53 removal subdivided regarding their belonging to the specified GO category.

Next, we performed profiling analysis of mutp53-inactivated HaCaT cells. A large number of genes were up -1649- or down -1644- regulated, by using a relatively stringent cut-off *ratio* of 1.5-fold (Supplementary Table 3). A quick inspection of the genes identified known p53 targets, such as CDC20, Aurora KinaseA and B, Chek1, Topo IIα, CyclinB1 and B2, CyclinA, CDC2, p57/Kip2. G2/M genes are normally repressed upon DNA-damage in cells harboring wt p53, and regulated in an opposite way by indirect recruitment by mutp53 [29, 30]. We validated the profiling results by qRT-PCR (Fig. 4A): essentially all genes changed expression according to expectations; the degree of variation was greater in qRT-PCRs with respect to profiling data, which is a common finding, indicating that we underscored the effect of mutp53 removal in our profiling analysis. The overlap between the mutp53 locations and gene expression analysis is robust -15%- but not absolute (DD, RM, unpublished). Some genes might be indirectly affected by mutp53 removal, and some of the targets are oblivious of its removal, at least in growing HaCaT cells. GO categorization identified *cell cycle*, as well as *DNA* and *RNA metabolisms* and *response to DNA-damage* as robustly enriched in the upregulated cohorts (Figure 4B); in the downregulated genes, p values were less significant with *sterol biosynthesis* and *keratinocyte differentiation* being somewhat enriched. Examples of genes of up and down-regulated categories are shown in Figure 4C.

## DISCUSSION

Our work addresses a debated topic concerning the mechanisms of action of GOF mutant p53 and p63. We found that (i) mutp53 HaCaT alleles are pro-growth and mutp53 have thousands of binding sites in the human genome;(ii) they affect gene expression profoundly, both by binding with p63 to consensus elements and by being tethered by other TFs to their locations.

Mice deficient for *TP53* develop tumors resembling SCC after UV irradiation, and other models harboring mutp53 alleles have aggressive features in their epithelial tumors, including increased capacity to metastatize. Hence the hypothesis that certain mutations are GOF has visibly gained ground [10,11]. Specific mutations of p53 alleles are a hallmark of SCC in humans [7]: although HaCaT cells are not tumorigenic *in vivo*, they harbour two alleles that are routinely found in SCC, in addition to large amount of a fully functional ΔNp63α, the most abundant isoform found in human keratinocytes: stable p63 inactivation in HaCaT, in fact, was impossible, presumably because it is required for cellular growth. Instead, inactivation of mutp53 led to suboptimal growth rates and a large change in gene expression, as shown in previous experiments [31], making the system suitable to study p63 in the presence of high amounts of mutp53.

**Figure 5:**
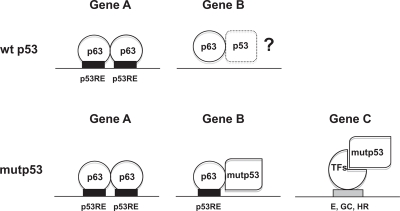
Models of p63 and mutp53 binding to different classes of promoters

Mechanistically, two models were proposed: (i) mutp53 sequesters tumor suppressors, including p63, in inactive complexes, or it inhibits its DNA-binding capacity [15, 17]; (ii) mutp53 acts as a *bona fide* transcription factor with a “deviant” specificity, through unrelated TFs, or the related p63/p73 [18-22]. Widespread inhibition of p63 DNA binding by p53 DNA-binding mutants seems to be ruled out by our experiments: >3400 p63 locations are retrieved in a cellular context with very high levels of tumor-type mutp53, up to 20-fold excess with respect to p63 [15]. The same conclusion is reached by analyzing gene expression profilings of overexpressed mutp53 in p53 null cells: Neilsen et al. find binding of mutp53 to 6 overexpressed genes [23]. Thus, at the heart of the mutp53 strategy there are interactions with p63, and with other TFs. Interestingly, the p63 locations are partially different from the ones found in normal keratinocytes [28], with >1700 “new” sites, showing an enrichment of mutp53 coresidency. In summary, the two previous scenarios for mutp53 GOF function, both involving tethering to DNA regulatory elements either with p63 (or p73), or *via* other TFs, are operational (Fig. 5). We attempted to “measure” the two mechanisms, and the second appears to be prevalent, but one needs to be very cautious and aware of the bias related to the antibodies used in the analysis: this is particularly relevant for mutp53, since DO1 is oblivious of the mut53 short isoforms very recently described by the lab of JC Bourdon in HaCaT [25]. The picture is therefore likely to be more complex.

The most abundant TFBS scored in the p63 peaks is indeed the p53/p63 RE, as found in Pscan analysis and in the stringent *de novo* motif discovery by Weeder, confirming that DNA-binding is direct through sequence-specific contacts and that p63 is functional. Our Weeder-derived logo incorporates the core central tetranucleotide, CANG from Chip on chip analysis of p63 and p73 [32, 33] and CNTG from SELEX [34]; it does provide additional information in the flankings, and the retrieved decamer is, to the best of our knowledge, the most detailed p63/p53 matrix characterized so far. In the mutp53/p63 *loci*, the p53/p63 logo is also obvious, both in Pscan and *de novo* motif discovery; the precision of the ChIP-Seq technique allows us to conclude that the two TFs, which are bound within 50 bp, share the same DNA motif. If anything, tandem elements with variable spacing are enriched in the p63, but not mutp53/p63 cohorts: in the latters, it is unclear whether mutp53 contacts with DNA have a different specificity, or lack it. In general, these data are consistent with a model whereby heteromers, formed by the observed increased affinity of mutp53 for p63 [12-14], and/or higher protein concentrations, are steered to DNA by the p63 sequence-specific capacity. Another aspect that has not been investigated so far is the reciprocal interplay between p63 and wt p53 after DNA-damage in normal cells (Fig. 5): one could imagine, in fact, that the binding of p63 might serve as a “guide” for an activated p53 to find its locations, or at least some of them, during the stress response.

As to tethering of mutp53 *via* unrelated TFs, the lack of a single, clearly recognizeable logo with *de novo* motif analysis strongly indicates that multiple TFs are involved. Even allowing a high degree of freedom, so that “non canonical” sites could be scored [35], we could not come up with any enriched motif. Among the TFBS previously reported to be guiding mutp53 binding, the NF-Y, E2F and NFkB sites [18-20] are statistically enriched in the profiling data (Supplementary Fig. 4). In the ChIP-Seq dataset, we do find the Estrogen Receptor, whose reciprocal interplay with mutp53 is well documented in other epithelial contexts, for example in breast cancer cells [36]. Note that HaCaT do not express ERs (EM, RM, unpublished), but an identical DNA motif is shared with other nuclear receptors, including VDR, recently shown to be enriched in ChIP on chip analysis of SKbR3 cells carrying the -175 p53 GOF mutant [21]. Therefore, our data suggest that mutp53 targets nuclear receptors. GC boxes, E-boxes, AP2 predominate in ChIP-Seq and profilings. E boxes are recognized by a plethora of HLH B-Zip proteins, some of which are known to play a role in epithelial cancer, including in the skin [37]. GC boxes are often overrepresented in many such studies, not least because promoters are embedded in CpG islands: this box is targeted by zinc fingers TFs belonging to the large Sp1 and KLFs family, numbering over 20 members [38]. We have recently detailed that one of these -KLF4- is targeted by mutp53, through p63 [22]. A large body of genetic, biochemical and histopathological evidence points to disregulation of KLF4, KLF5 and KLF6 as important in the progression of epithelial tumors. The lack of previously identified motifs -NFkB, NF-Y, E2F- in the ChIP-Seq dataset is not surprising, considering that the mutant p53 alleles and the cellular context used here are different: this raises the possibility that the set of TFs tethering mutp53 might be specific for mutp53 alleles and/or for a particular cell-type, whether in immortalized or transformed conditions.

As a whole, the p63 preferred GO categories are a variation of *morphogenesis, tissue* and *development* themes, as expected from a master ectodermal regulator; clustering of the normal keratinocytes and HaCaT p63 locations tells a similar story, with targets in the *RNA metabolism* and *transcription* category, which we previously reported. Mutp53, however, visibly changed the configuration, since the mutp53^+^, as well as the common mutp53/p63^+^, are shifted toward and enrichment of signaling, cell-cycle regulation and metabolic terms. As for single pathways, those that were previously pinpointed -FGF-R, EGF-R, Wnt, Notch- are confirmed. At least one previously unappreciated group of genes emerged: RARα, RARγ and RXR genes are targeted at multiple locations both by p63 and mutp53. Interestingly, genetic experiments in mice using a dominant negative RARα expressed in basal keratinocytes *via* the K14 promoter caused inhibition of endogenous RARα and RARγ with greatly diminished p63 levels: this led to a dramatic skin phenotype very similar to the one found in p63 KO mice [39]. Given the general anti-proliferative properties of RARs, including in the skin [40], the intersection of p63/mutp53 with RARs is certainly worth more exploration in the future, particularly in tumors.

In summary, our data support the idea that p53 mutants do affect growth by altering gene expression *via* specific binding to discrete DNA elements, in promoters and elsewhere, either through p63, or selected classes of TFs. Furthermore, the normal p63 regulome is altered, and mutp53 partially diverts p63 activity to locations not normally seen in normal keratinocytes. Our work should be extended to different cellular contexts, in tumorigenic cells carrying different GOF mutp53 and splicing isoforms of p63 as well as the related p73.

## MATERIALS AND METHODS

### Cells and Infections

Human HaCat keratinocytes were maintained in DMEM supplemented with 10% FCS, 2 mM glutamine and 100 U penicillin/streptomycin. The growth characteristics were obtained by counting the cells in the hemacytometer at the indicated time points. For UVB irradiation, 80% confluent HaCaT cells was replaced by PBS and the cells were exposed to various concentrations (20mJ/cm_2_, 40mJ/cm_2_) of UVB light emitted by MacroVue UV-20 (Hoefer, USA) with the peak at 320 nm. After removal of PBS the growth medium was added and the cells were incubated for 12 h at normal conditions and then harvested with cell scraper including those floating in medium. PBS-treated cells without UVB treatment served as negative control.

For the cell cycle analysis 10^6^ cells were washed once in PBS and then fixed with 70% cold ethanol. After this one wash in 1% BSA in PBS was performed and the cells were stained for 30' in 1ml of PI solution (20μg/ml Propidium Iodide, 10μg/ml Rnase solution in PBS). The data were acquired by FACS within 2 h after staining. TUNEL assay for detection of apoptosis was performed according to the manufacturer's protocol (Roche Applied Science, Germany). The cells were fixed for 1h in 4% Paraformaldehyde in PBS, rinsed with PBS and permeabilised for 2' in 0,1% Triton X-100 in 0,1% sodium citrate. After 2 washes with PBS cells were stained with 50 μl of TUNEL reaction mixture and then with DAPI and the cells were immediately analysed under a fluorescence microscope.

For the lentivirus production one day before 293T cells were split at the density of 5*106 cells per plate using 20 ml of DMEM medium supplemented with 10% FBS. For the 3 plasmid system the following DNA mix was prepared: 20μg lentiviral vector, 10μg VSVG, 15μg Δ3.8. In the tube with DNA, 400μl of 1,25 MCaCl_2_ and 1,5ml of H_2_O were added; 2ml of 2X HBS (280mM NaCl, 50mM Hepes, 1,5mM Na_2_HPO_4_, pH 6,95) were added. The transfection mixture was kept 12h after which the medium was changed. After 36h, the viral supernatant was harvested and filtered through a 0,45μm filter. The lentiviral infection of HaCaT cells were performed by double spinoculation of 70% confluent cells (1h centrifugation, 2000 rpm, with a 5h interval) in the presence of 2 μg/ml polybrene. 72 h after infection puromycine (Sigma, USA) was added in the medium for selection. For p53 knockdown in the HaCaT cells, the shp53 pLKO.1 puro plasmid (Addgene, USA) was used and shscramble pLKO.1 puro plasmid was used as control. After 3 weeks in the selection medium, stable clones were pooled.

Western blot analysis was perfomed according to standard procedures with whole cell extracts with DO1 anti-p53, anti-p63 (Genespin, Italy), anti-vinculin (Sigma, USA), anti-cleaved Caspase 8 (Cell Signaling Technology, USA) and anti-PARP (Santa Cruz, USA) antibodies.

### ChIP and ChIP-Seq

ChIP was carried out as previously described (21). In brief, about 5 mg of chromatin (equivalent to 18 × 150 mm dishes with cells at 80% confluence) were used in IP experiments, with either mouse monoclonal anti-p53 (DO1), or rabbit polyclonal anti-p63 antibodies (Genespin, I). Each chromatin set was divided into 10 aliquots, which in turn were independently IPed using 10μg of the appropriate antibody. In parallel, 500 μg of chromatin were IPed with 10 g of mouse monoclonal anti-Flag antibody (Sigma) as a control. ChIP-enriched and their Unbound fractions were recovered and subject to crosslink reversal, proteinase K digestion, phenol/chloroform extraction, DNA precipitation and quantitation. Single ChIP-enriched DNA samples were then tested by qPCR to assess enrichment on known targets of either p53 and/or p63. p63 enrichment (mean ± SD) on Myoneurin promoter and C40 enhancer were 2.76 ± 0.46 and 49.88 ± 18.61, respectively; p53 enrichment on LEF1 upstream region were 1.74 ± 0.34 and 7.05 ± 2.12. ChIP-enriched DNAs, as well as half of the corresponding Unbound DNAs, were pooled together, precipitated and quantitated. 50 ng of each ChIP-enriched or Unbound DNA were then converted into a library suitable for high-throughput sequencing using an Illumina Genomic Analyzer following the manufacturer's istructions. Before sequencing, amplified ChIP-samples were tested in parallel to amplified Unbound, pre-amplification ChIP-enriched, and pre-amplification Unbound DNAs, to score for enrichment. Sequence reads were mapped to the masked human genome sequence (assembly GRCh37, retrieved from the UCSC genome browser database (38) using the Seqmap tool (39). Matched was performed by allowing at most two mismatches at any position of the reads. Trimming unmapped reads at the 3'end led to marginal improvements in the number of mapped reads and this step was therefore skipped. Only reads mapping to a unique position on the genome were considered for further analysis. This resulted in about 10 million uniquely mapping reads for each of the two mutp53 experiments (IP and input) and in about 4 million for each of the two p63 experiments. In each experiment, uniquely mapped reads were then extended by 300 bps along the 5'->3' direction. This produced, for each ChIP or input sample, a base pair by base pair coverage map of the genome, that is, giving for each base pair the number of extended sequence reads that contained it. Only base pairs covered by reads mapping on both strands were considered valid for further analysis. Enrichment was then calculated in each valid base pair by comparing, for each IP experiment, the coverage in the experiment to the coverage in the respective input used as expected value, and computing an enrichment p-value with a negative binomial distribution. Enriched regions were then defined as regions consisting of consecutive base pairs characterized by calculated p-values smaller than 0.01 and not interrupted by a gap of 100 bps or more non valid or with a p-value greater than 0.01. The p-value associated with each of the enriched regions was defined as the minimum p-value among the base pairs belonging to the region. Regions shorter than 150 bps were then discarded regardless of the p-value. The p-value associated with the remaining regions was then used to compute the false discovery rate (FDR) with the Benjamini-Hochberg correction. This resulted in 7136 regions for p53 and 3422 for p63 with FDR lower than 0.01.

To further validate the predicted regions we applied the MACS tool (40) to the same datasets, with default parameters. About 95% of our predicted regions in both mutp53 and p63 experiments were also found as significantly enriched by MACS at p-value 10^−6^ (roughly corresponding to our False Discovery Rate of 0.01).

### RNA Profiling

Total RNA was extracted using RNeasy Mini kit (Qiagen, D) according to the manufacturer's protocol. For qPCR analysis, 1μg of RNA was reverse-trascribed using Reverse Transcription System (Promega, USA). The expression level for each gene was normalized with GAPDH. The list of the primers used for qPCR is shown in Supplementary 4. For profiling, RNA quality was assessed using an Agilent 2100 Bioanalyzer (Agilent Technologies, USA). 500 ng of total RNA was synthesized to biotinylated cRNA using the Illumina RNA Amplification Kit (Ambion, USA). 750 ng cRNA was hybridized for 18 h to HumanHT12 v. 3.0 Expression BeadChips (Illumina USA) according to the protocol provided by the manufacturer. Hybridized chips were washed and stained with streptavidin-conjugated Cy3 (GE Healthcare, USA). BeadChips were dried and scanned with an Illumina BeadArray Reader (Illumina Inc.) and analyzed with the Illumina BeadStudio v. 3.1.3.0 software. The quantile normalization algorithm was applied on the data set to correct systematic errors. Background was subtracted. For differential expression analysis, three technical replicates of each sample were grouped together and genes with a detection of p-value <0.01, corresponding to a false-positive rate of 1%, were considered as detected. Differently expressed genes were selected with Diff Score cutoff set at ±30, corresponding to a P-value of 0.001.

## Supplementary Tables and Figures
















