# Supplementary material for: Gain-of-function p53 mutants have widespread genomic locations partially overlapping with p63
Source: Oncotarget. 2012 Feb 22;3(2):132–43. doi: 10.18632/oncotarget.447 (PMC3326644; doi:10.18632/oncotarget.447)
Supplement: Supplementary file 4 [file oncotarget-03-132-s004.ppt]

## Slide 1
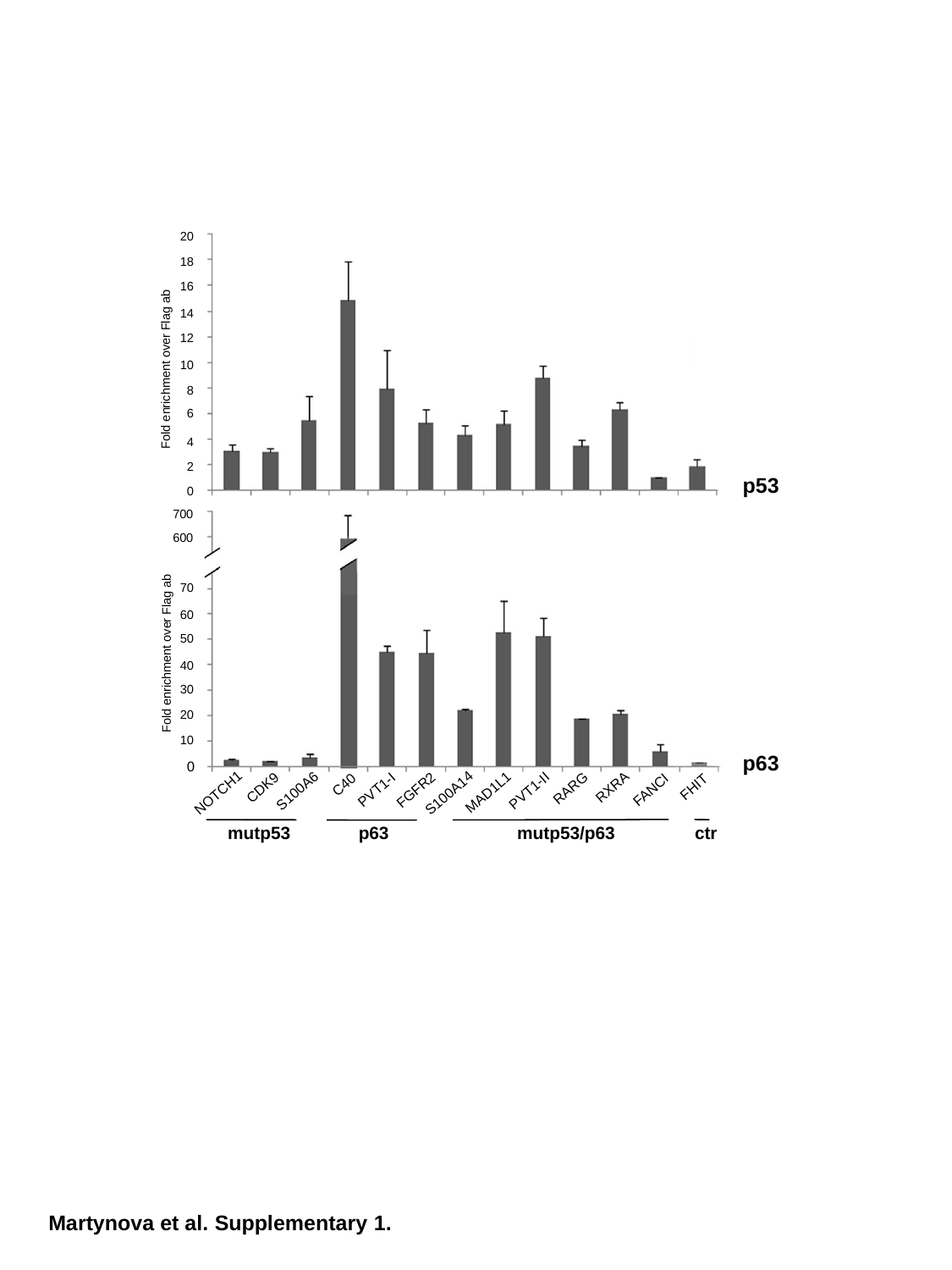

20
18
16
14
12
10
Fold enrichment over Flag ab
8
6
4
2
p53
0
700
600
70
60
50
Fold enrichment over Flag ab
40
30
20
10
p63
0
C40
FHIT
RXRA
CDK9
RARG
FANCI
PVT1-I
FGFR2
PVT1-II
S100A6
MAD1L1
NOTCH1
S100A14
mutp53
p63
mutp53/p63
ctr
Martynova et al. Supplementary 1.
