# Supplementary material for: Gain-of-function p53 mutants have widespread genomic locations partially overlapping with p63
Source: Oncotarget. 2012 Feb 22;3(2):132–43. doi: 10.18632/oncotarget.447 (PMC3326644; doi:10.18632/oncotarget.447)
Supplement: Supplementary file 5 [file oncotarget-03-132-s005.pdf]

p63 positive targets.

Notch1	p63/mutp53	(Nguyen BC et al., *Genes Dev.* 2006; 20: 1028-42)
p21	p63	(Westfall MD et al., *Mol Cell Biol.* 2003; 23: 2264-76)
VDR	p63	(Kommagani R et al., *Oncogene* 2006; 25: 3745-51)
TP63	p63/mutp53	(Antonini D et al., *Mol Cell Biol.* 2006; 26: 3308-18)
KLF4	p63/mutp53	(Cordani N et al., *Oncogene* 2011; 30: 922-32)
S100A2	p63	(Kirschner RD et al., *Nucl Acids Res.* 2008; 36: 2969-80)
SPATA18	p63	(Bornstein C et al., *Mol Cell Biol.* 2011; 31: 1679-89)
IRF6	p63	(Moretti F et al., *J Clin Invest.* 2010; 120: 1570-7)
REDD1	mutp53	(Ellisen LW et al., *Mol Cell* 2002; 10 :995-1005)
WNT4	p63	(Osada M et al., *BBRC* 2006; 339: 1120-8)
BPAG1	p63	(Osada M et al., *J Invest Dermatol.* 2005; 125:52-60)
ATM	mutp53	(Craig AL et al., *Mol Cancer* 2010; 9:195)
FAS/CD95	p63	(Schilling T et al., *BBRC* 2009; 387: 399-404)
CDH3	p63/mutp53	(Shimomura Y et al., *Development* 2008; 135:743-53)
TRAF4	p63/mutp53	(Gu X et al., *Cancer Biol Ther.* 2007; 6:1986-90)
CD44 KRT6 KRT14, KRT19	p63/mutp53	(Boldrup L et al., *J Pathol.* 2007;213:384-91)
DLX3/4	p63/mutp53	(Radoja N et al., *Development* 2007; 134: 13-8)
FGFR2	p63	(Sayan AE et al., *BBRC* 2010; 394:824-8)
JAG2	p63/mutp53	(Candi E et al., *PNAS* 2007; 104:11999-2004)
DHRS3	p63	(Kirschner RD et al., *Cell Cycle* 2010; 9: 2177-88)

Martynova et al. Supplementary Fig. 2
