# Supplementary material for: Gain-of-function p53 mutants have widespread genomic locations partially overlapping with p63
Source: Oncotarget. 2012 Feb 22;3(2):132–43. doi: 10.18632/oncotarget.447 (PMC3326644; doi:10.18632/oncotarget.447)
Supplement: Supplementary file 6 [file oncotarget-03-132-s006.ppt]

## Slide 1
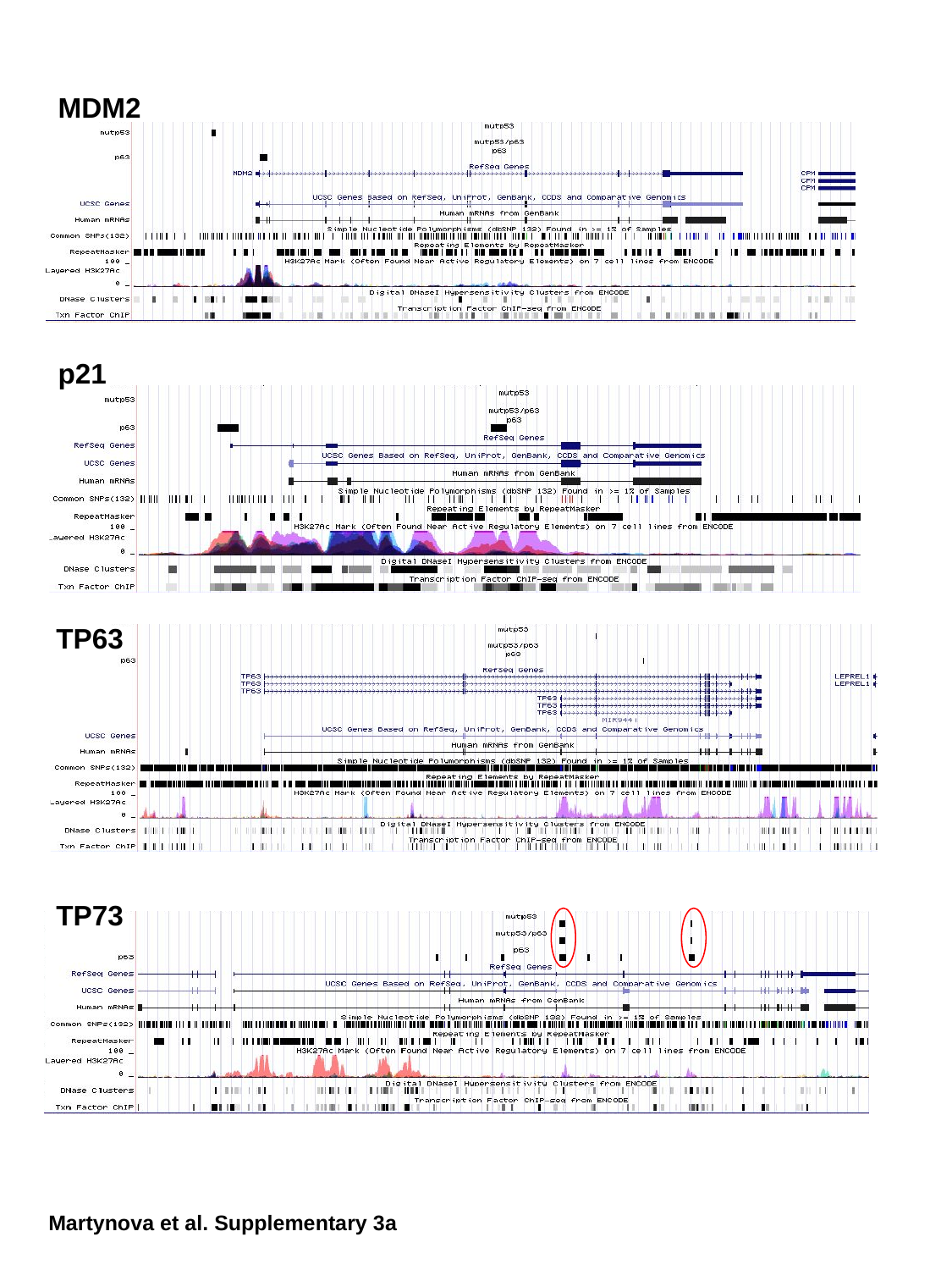

MDM2
p21
TP63
TP73
Martynova et al. Supplementary 3a

## Slide 2
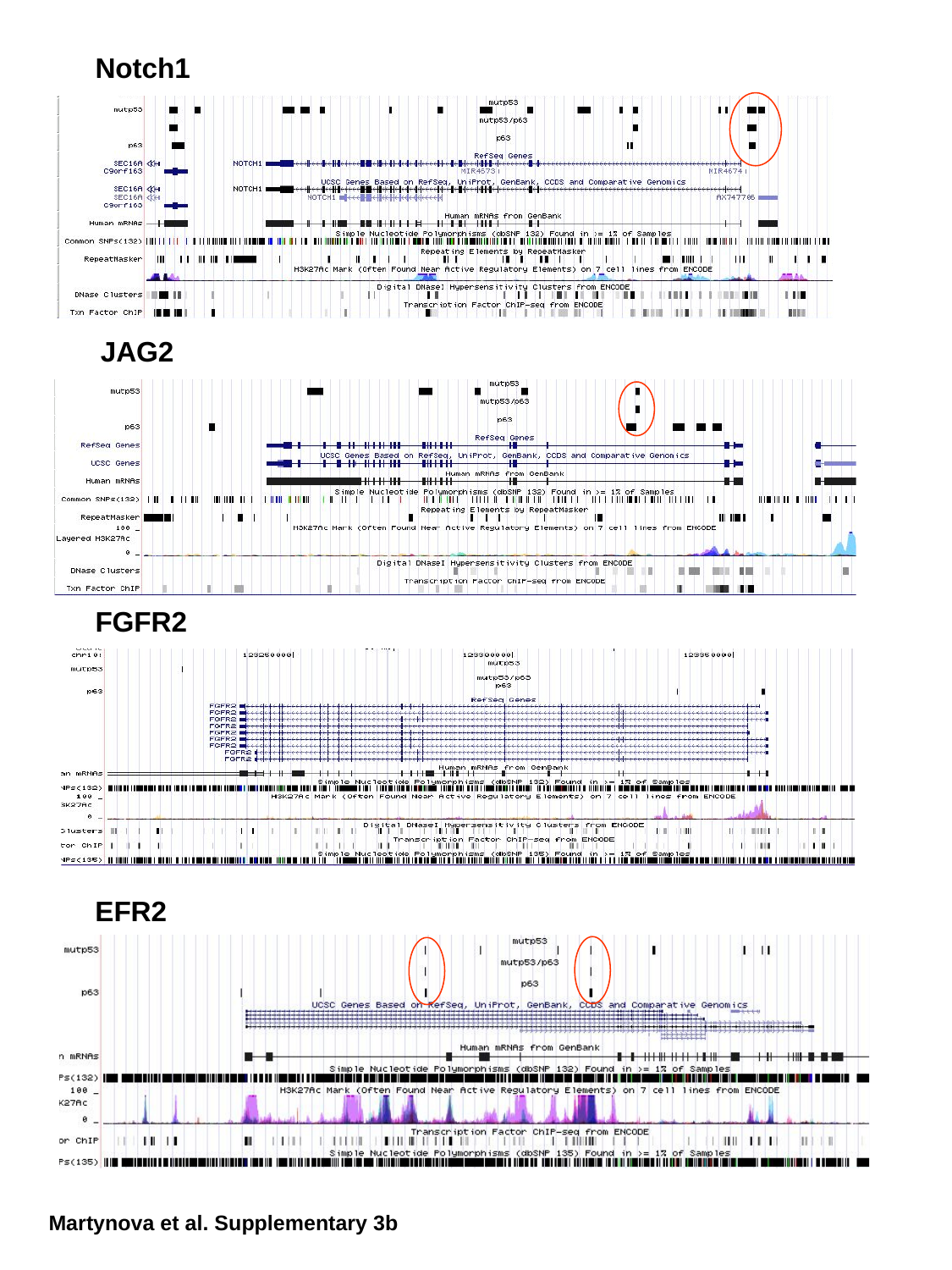

Notch1
JAG2
FGFR2
EFR2
Martynova et al. Supplementary 3b

## Slide 3
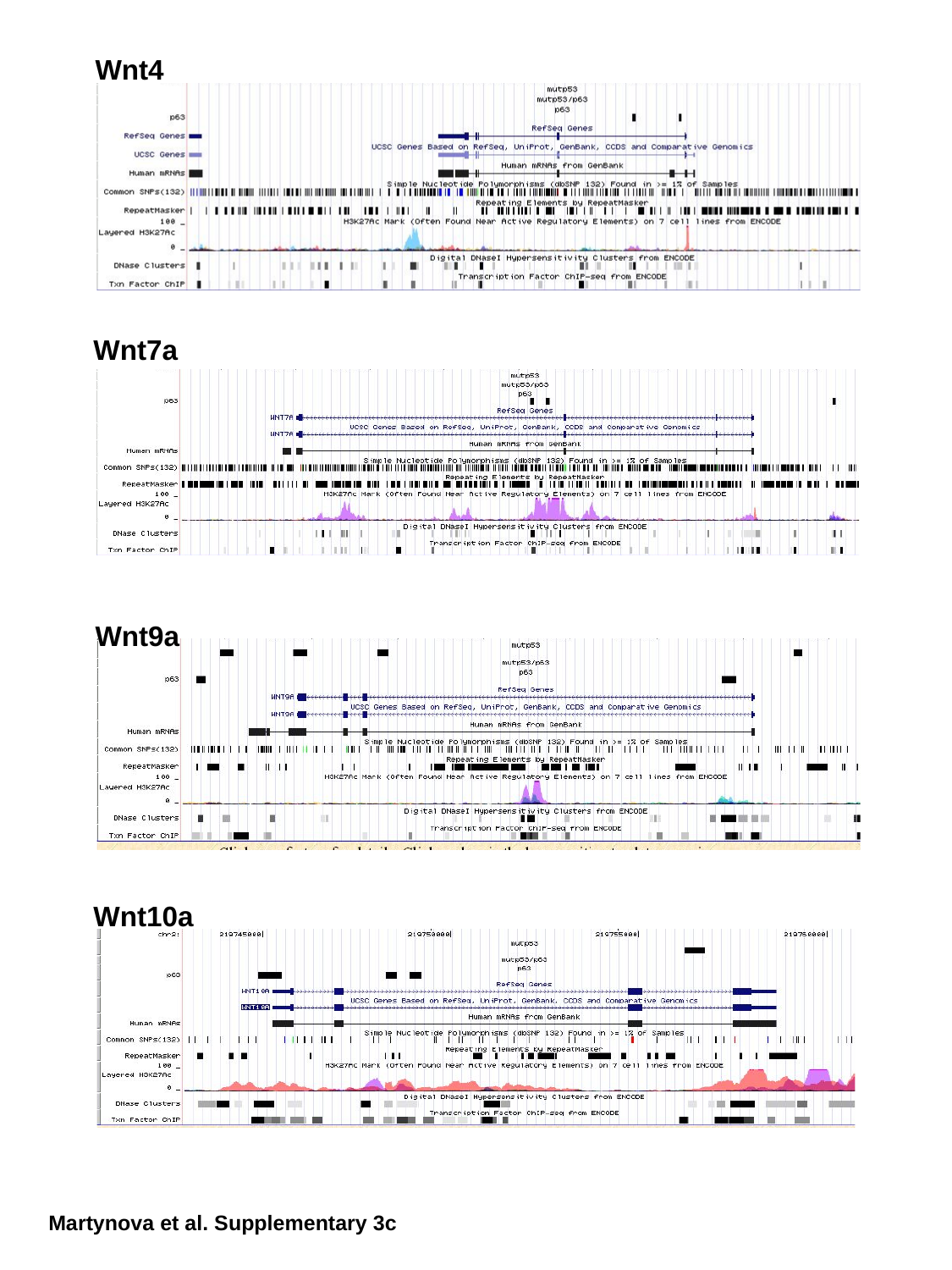

Wnt4
Wnt7a
Wnt9a
Wnt10a
Martynova et al. Supplementary 3c

## Slide 4
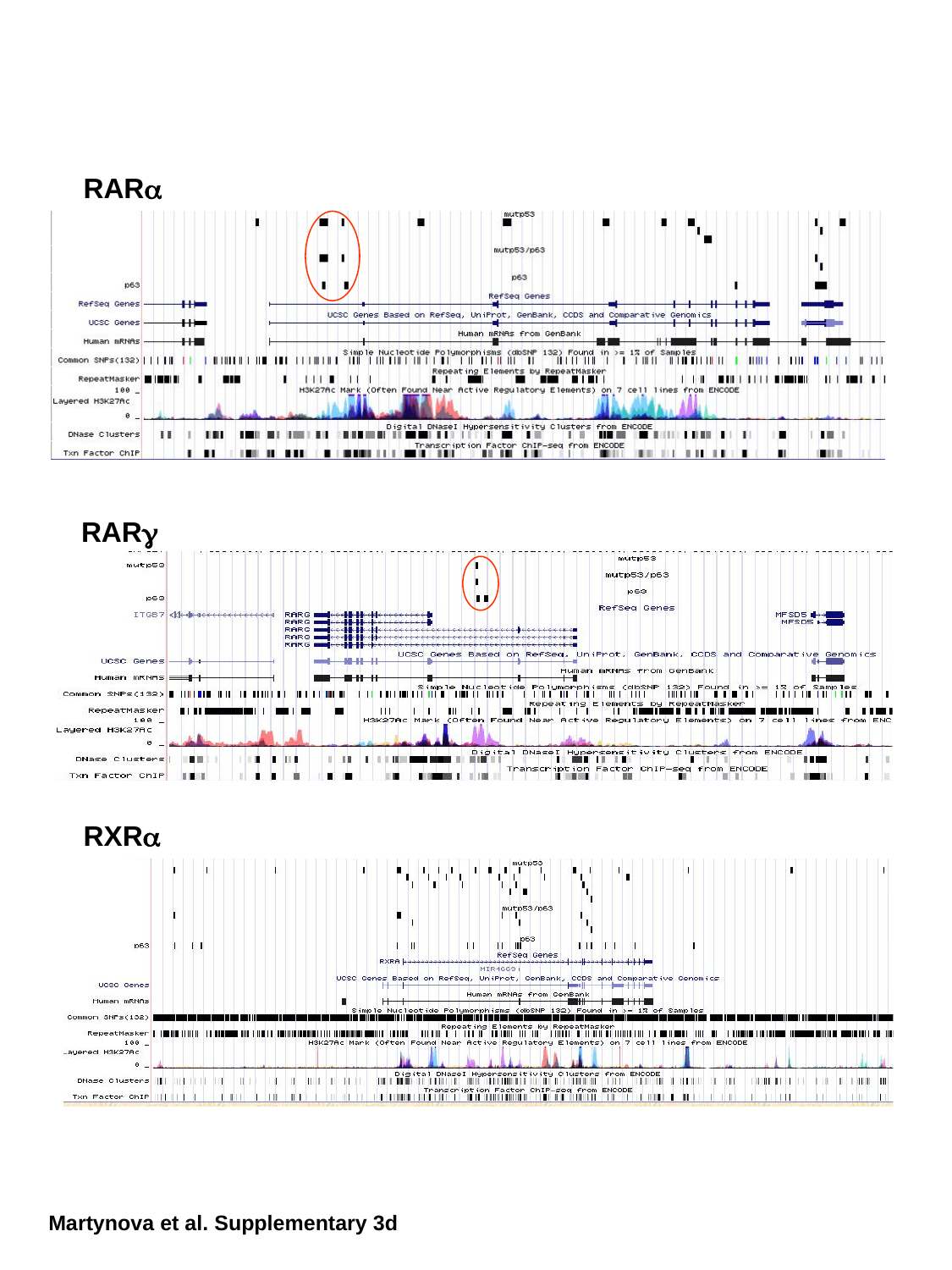

RARα
RARγ
RXRα
Martynova et al. Supplementary 3d

## Slide 5
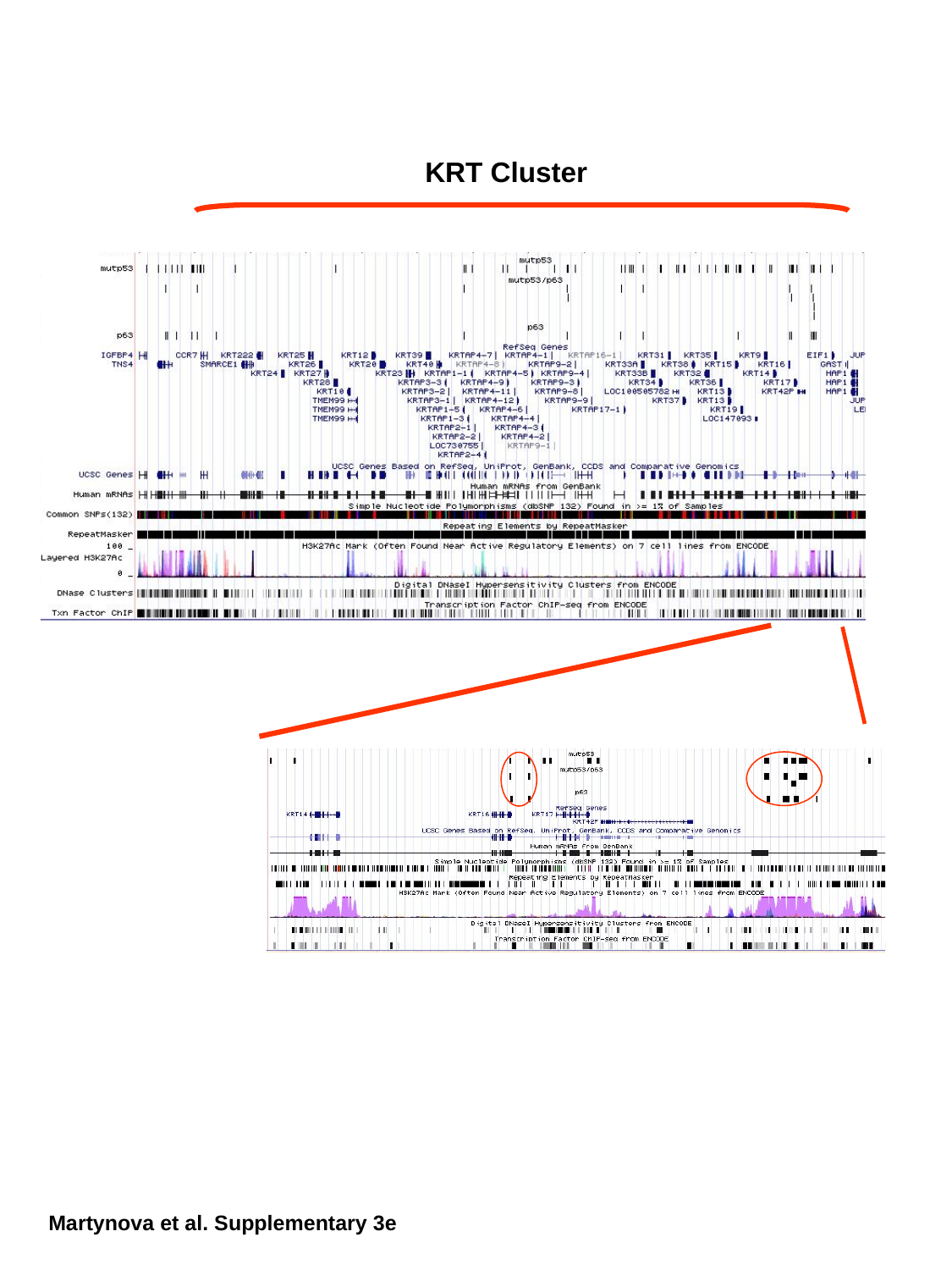

KRT Cluster
Martynova et al. Supplementary 3e
