# Supplementary material for: Gain-of-function p53 mutants have widespread genomic locations partially overlapping with p63
Source: Oncotarget. 2012 Feb 22;3(2):132–43. doi: 10.18632/oncotarget.447 (PMC3326644; doi:10.18632/oncotarget.447)
Supplement: Supplementary file 7 [file oncotarget-03-132-s007.ppt]

## Slide 1
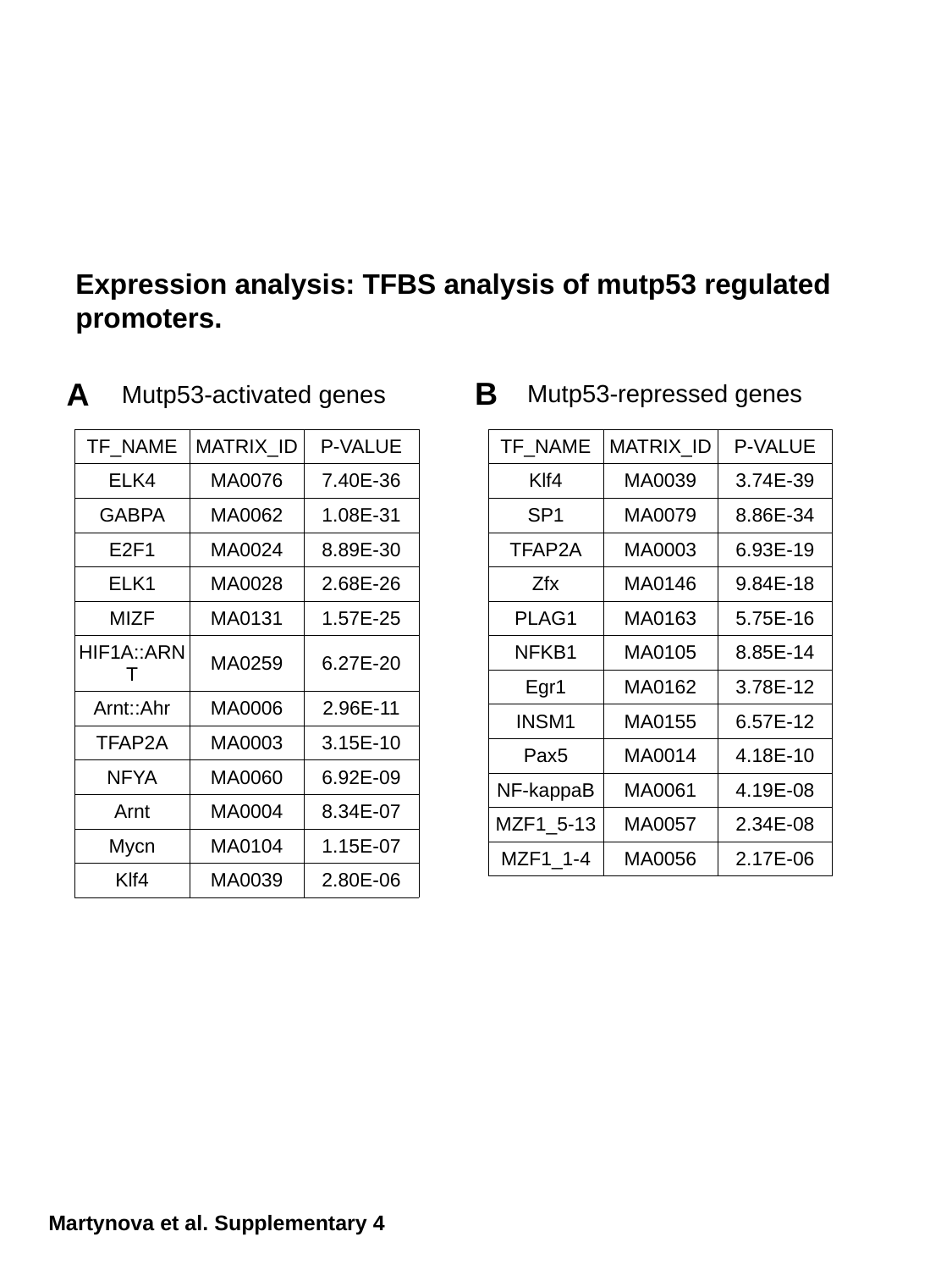

Expression analysis: TFBS analysis of mutp53 regulated promoters.
B
A
Mutp53-repressed genes
Mutp53-activated genes
| TF\_NAME | MATRIX\_ID | P-VALUE |
| --- | --- | --- |
| ELK4 | MA0076 | 7.40E-36 |
| GABPA | MA0062 | 1.08E-31 |
| E2F1 | MA0024 | 8.89E-30 |
| ELK1 | MA0028 | 2.68E-26 |
| MIZF | MA0131 | 1.57E-25 |
| HIF1A::ARNT | MA0259 | 6.27E-20 |
| Arnt::Ahr | MA0006 | 2.96E-11 |
| TFAP2A | MA0003 | 3.15E-10 |
| NFYA | MA0060 | 6.92E-09 |
| Arnt | MA0004 | 8.34E-07 |
| Mycn | MA0104 | 1.15E-07 |
| Klf4 | MA0039 | 2.80E-06 |
| TF\_NAME | MATRIX\_ID | P-VALUE |
| --- | --- | --- |
| Klf4 | MA0039 | 3.74E-39 |
| SP1 | MA0079 | 8.86E-34 |
| TFAP2A | MA0003 | 6.93E-19 |
| Zfx | MA0146 | 9.84E-18 |
| PLAG1 | MA0163 | 5.75E-16 |
| NFKB1 | MA0105 | 8.85E-14 |
| Egr1 | MA0162 | 3.78E-12 |
| INSM1 | MA0155 | 6.57E-12 |
| Pax5 | MA0014 | 4.18E-10 |
| NF-kappaB | MA0061 | 4.19E-08 |
| MZF1\_5-13 | MA0057 | 2.34E-08 |
| MZF1\_1-4 | MA0056 | 2.17E-06 |
Martynova et al. Supplementary 4
