# Supplementary material for: Gain-of-function p53 mutants have widespread genomic locations partially overlapping with p63
Source: Oncotarget. 2012 Feb 22;3(2):132–43. doi: 10.18632/oncotarget.447 (PMC3326644; doi:10.18632/oncotarget.447)
Supplement: Supplementary file 8 [file oncotarget-03-132-s008.pdf]

qRT-PCR primers

p53_for	5'-AAGGAAATTTGCGTGTGGAGT-3'
p53_rev	5'-AAAGCTGTTCCGTCCCAGTA-3'
gapdh_for	5'-GCCTCAAGATCATCAGCAATGC-3'
gapdh_rev	5'-CCACGATACCAAAGTTGTCATGG-3'
p63_for	5'-GAAGAAAGGACAGCAGCATTGAT-3'
p63_rev	5'-GGGACTGGTGGACGAGGAG-3'
PHYHD1_for	5'-CTTAGAAGCCGCCCAGTG-3'
PHYHD1_rev	5'-CAGCCACTATCTCGCCAATC-3'
TSC22D3_for	5'-AGCGTGGTGGCCATAGAC-3'
TSC22D3_rev	5'-CACGCTCTAGCTGGGAGTTC-3'
TNFSF9_for	5'-CGAGCTTTCGCCCGACGAT-3'
TNFSF9_rev	5'-TGGGTCACTGTACCAGCTCA-3'
ECM1_for	5'-CCCACCTCTGAGTGTCCAGT-3'
ECM1_rev	5'-AGCTGCCTCTGTCCTGTAGC-3'
TGM5_for	5'-AGCCTGCATACACCTTCCCTTC-3'
TGM5_rev	5'-AACGCTGTGTCCTGCCAGAATG-3'
KRT16_for	5'-GCCGACCTGGAAGTGAAGAT-3'
KRT16_rev	5'-GCTGCGCATTCTCAATGG-3'
p57_for	5'-TCGCTGCCCGCGTTTGCGCA-3'
p57_rev	5'-CCGAGTCGGTGTCCACTTCGG-3'
FANCI_for	5'-GAGCAACAATGGACCAGAAG-3'
FANCI_rev	5'-AGTGCTCCAGCAACTTTTCC-3'
AURKA_for	5'-GTCACAAGCCGGTTCAGAAT-3'
AURKA_rev	5'-TTTGATGCCAGTTCCTCCTC-3'
E2F2_for	5'-AAGTGCATCAGAGTGGATGGCCT-3'
E2F2_rev	5'-AATGAACTTCTTGGTGAGCAGCCC-3'
CCNB2_for	5'-CAGTTCCCAAATCCGAGAAA-3'
CCNB2_rev	5'-TCTGAGACAAGCAGGAAGCA-3'
Top2A_for	5'-ACCATGGAAGTGTCACCATTGCAG-3'
Top2A_rev	5'-CTGGGTCACTAATTCCACAGAACC-3'
RAD51AP1_for	5'-GGCTCCGAAGTCTGGTTTT-3'
RAD51AP1_rev	5'-CTGTCAGAGTGGTCAAACTGTG-3'
Oip5_for	5'-GAAGCGCCCTTCCTAGTTG-3'
Oip5_rev	5'-CTGGAAAGGCAGAAGTGACC-3'
CCNB1_for	5'-CACTTCCTTCGGAGAGCATC-3'
CCNB1_rev	5'-CAGGTGCTGCATAACTGGAA-3'
CCNA2_for	5'-TATTGCTGGAGCTGCCTTTC-3'
CCNA2_rev	5'-CTCTGGTGGGTTGAGGAGAG-3'
TMEM20_for	5'-GCAAGACGTCCATGCTGTAG-3'
TMEM20_rev	5'-GGCGGTAGAACCAAGGACTC-3'
CDCA3_for	5'-CCTATGAAGACCAGCAGTGG-3'
CDCA3_rev	5'-GTACCCAGAGGCAAGTCCAA-3'

qPCR ChiP primers

NOTCH1_for	5'-GCAGTAGAAGGAGGCCACAC-3'
NOTCH1_rev	5'-GTGTGTCAACGGCTGGACT-3'
CDK9_for	5'-AGAAGCGGAAGGTGAAGGA-3'
CDK9_rev	5'-CAGAAGAAGTCGTGGTTGAGG-3'
S100A6_for	5'-CACCCACACACCCCAATAAT-3'
S100A6_rev	5'-GTGCTCTCTCCTTCCCCTTC-3'
C40_for	5'-GGGGATGCTTTTACTCGTTCCA-3'
C40 _rev	5'-TCCCCTGTCTTTCCTCCCAAA-3'
PVT1-I_for	5'-CGCAGGGACAGGTAGATCAT-3'
PVT1-I_rev	5'-CGAGTCAGCCCAGAGGAAT-3'
FGFR2_for	5'-AGGTCGTATCCAAAACAAGCAA-3'
FGFR2_rev	5'-GGCAGTTCAGTGGTGTGACTC-3'
S100A14_for	5'-GAGCACTTGCTTGGGAAGTC-3'
S100A14_rev	5'-CTGACCCCTTCTGAGCTACG-3'
PVT1-II_for	5'-CCTGTTTTGCCTGTTTACCC-3'
PVT1-II_rev	5'-AGTCAGCCCAGAGGAATGC-3'
RARG_for	5'-GGCTTCCCTTCTCTTTCATCA-3'
RARG_rev	5'-GCGTGCGTGTGTGTTCAT-3'
RXRA_for	5'-CCATTCTGCTTCCTCCTCCT-3'
RXRA_rev	5'-CTGTTTCCACACCCAGTCCT-3'
FANCI_for	5'-AGGAGGGAAGCTGAACCT-3'
FANCI_rev	5'-CGGGGTGTACCATGAAGACT-3'
FHIT_for	5'-GAGTGGGGAAATGAAACGAA-3'
FHIT_rev	5'-GGGCATAAATCGCACAATCT-3'

Martynova et al. Supplementary Fig. 5
